# Supplementary material for: Utilization and factors associated with health facility delivery among women of reproductive age in rural Ethiopia: Mixed effect logistic regression analysis
Source: PLoS One. 2023 Jul 14;18(7):e0280660. doi: 10.1371/journal.pone.0280660 (PMC10348594; doi:10.1371/journal.pone.0280660)
Supplement: Attachment — Submitted filename: Response to Reviewers.docx [file pone.0280660.s002.docx]

Manuscript PONE-D-22-35754

PLOS ONE Response to Reviewers

Dear Dr. Anteneh Fikrie
Academic Editor
PLOS ONE

Thank you for giving me the opportunity to submit a revised draft of the manuscript “Utilization and Factors Associated with Health Facility Delivery among Women of Reproductive Age in Rural Ethiopia: Mixed effect logistic regression analysis.” for publication in the PLOS ONE journal. I appreciate the time and effort that you and the reviewer (Dr. Kebede Tefera) dedicated to providing feedback, and I am grateful for the insightful comments on and valuable inputs you have provided, which improved the manuscript. I have incorporated all of the suggestions made. Those changes are highlighted within the revised manuscript with track changes. Please see below, in the table, for a point-by-point response to the reviewers’ comments and concerns. **All line numbers refer to the revised manuscript file without tracked changes.**

| **Academic Editor comment** | **Response** |
| --- | --- |
| 1)Result section under Table 3. Educational status: Higher (AOR=20.42; 95%CI:2.76 - 151.11) The confidence interval is too wide perhaps this could be due to the smaller sample size. Thus, I suggested the authors to consider merging Higher educational status and Secondary educational status so that you can create a new variable secondary and above will be created and the issue of wider confidence interval will be hopefully solved. | Thank you for pointing this out. Revision have been made accordingly kindly find it in line number 172, and Table 3 |
| 2)Similarly, the authors considered Religion under statistical regression. What would be the implication if it was found to be statistically significant? | I am grateful for your concern here. Religion have been taken out of the regression model. |

| **Reviewer#1 comments** | **Responses** |
| --- | --- |
| 1) Line 69 at the period of the study in 2022, Ethiopia has 11 state and two cities (the eleven-state established as the South West Ethiopia Region was created on 23 November 2021). | Dear Dr. Kebede, I am really grateful for pointing this out. But the thing is the manuscript is based on secondary data (EMDHS2019). The survey was conducted based on the nine regions and two cities of Ethiopia from March 21, 2019, to June 28, 2019. |
| 2) Higher Educational status of AOR was too wide 20.42(2.76,151.11) it indicates that the precision of the estimate is low, meaning that there is a high level of uncertainty surrounding the true population parameter, thus it needs some correction | Thank you for pointing this out. This has been corrected, kindly look at line number 172,and Table3. |
| 3) Limitation of the study was not explained , so identifying and discussing the limitations, researchers can demonstrate their awareness of the scope and boundaries of their study, ensure transparency and objectivity in their reporting, and provide recommendations for future research to address the gaps or uncertainties. | Thank you for pointing this out. The limitation of the study and future recommendations have been mentioned from line number 230 up to 236. Kindly look at it. |

**Additional change made**: In the first draft of the manuscript there was an error in recoding the variable “Having ANC in the first trimester of pregnancy”. So, change has been made to correct it.

**NB**: Table 1-3, and figure 2 have been updated following the recoding of the variable’s educational status, and Having ANC in the first trimester of pregnancy

Looking forward to hearing from you at your earliest convenience.

Thank you for your consideration of this manuscript!

Sincerely,

**Birhan Ewunu Semagn**
